# Polyvictimization among a juvenile Portuguese sample

**DOI:** 10.1080/07853890.2021.1896152

**Published:** 2021-09-28

**Authors:** Telma C. Almeida, Catarina Ramos, Jorge Cardoso

**Affiliations:** ^a^Laboratório de Psicologia Egas Moniz (LabPSI-EM), Centro de Investigação Interdisciplinar Egas Moniz (CiiEM), Egas Moniz Cooperativa de Ensino Superior, Caparica, Portugal

## Abstract

**Introduction:**

Some adolescents experience more than one type of abuse [1] that can occur under different incidents during childhood and/or juvenile development [2]. Polyvictimization can be defined as the experience of four or more types of violence, including childhood neglect, psychological, physical or sexual abuse, witnessing violence [3]. In Portugal, studies on polyvictimization are very scarce [4]. The objectives of the current study are to analyse the prevalence of polyvictimization in a sample of Portuguese youth, to compare differences in polyvictimization between age and gender groups, and to verify the probability of occurrence of different types of victimisation among boys and girls.

**Materials and methods:**

The sample was composed of 849 participants, with 459 girls and 390 boys, and ages between 12 and 17 years old (*M* = 13.70, SD = 1.43). The study was conducted in Portuguese schools. The data collection was conducted according to ethical principles, with the authorisation of the schools, the parents, and the youth. Participants answered face to face to a sociodemographic questionnaire and to the Juvenile Victimisation Questionnaire (JVQ) that assesses to conventional crimes, child maltreatment, peer and sibling victimisation, sexual victimisation, and witnessing and indirect victimisation [4].

**Results:**

Over the last year, 67% (*n* = 388) of the participants had experienced at least one type of victimisation. The results reported differences in polyvictimization concerning age groups and not between gender groups. The difference between the group 12–14 years and the group 15–17 years was significant [*χ*^2^(3, *n* = 849) = 8.793, *p* = .032], and dependent on polyvictimization. Concerning gender groups, the difference in victimisation levels between boys and girls was not significant. However, the probability of occurrence of the various forms of victimisation was different between genders. Boys showed higher probability of occurrence of assault without weapon (OR = 0.430; *p* < .01; boys = 20.8%, girls = 14.2%), attempted assault (OR = 0.540; *p* < .01; boys = 18.5%; girls = 10.9%), nonsexual genital assault (OR = 0.241; *p* < .001; boys = 15.9%; girls = 4.4%), and burglary of family household (OR = 0.407; *p* < .05; boys = 6.2%, girls = 2.6%). However, girls revealed higher probability of occurrence of psychological/emotional abuse (OR = 1.672; *p* <.01; boys = 17.9%; girls = 26.8%).

**Discussion and conclusions:**

This research studies the polyvictimization in a Portuguese sample of youth. The results highlight the high prevalence of victimisation among young Portuguese youth. They also point out the differences between boys and girls concerning the types of victimisation and between the oldest and the youngest concerning polyvictimization. Our results are in line with some recent researches [5,6], and they can help to develop future psychosocial prevention programs.
